# “We’re checking them out”: Indigenous and non-Indigenous research participants’ accounts of deciding to be involved in research

**DOI:** 10.1186/s12939-016-0301-4

**Published:** 2016-01-16

**Authors:** Marilys Guillemin, Lynn Gillam, Emma Barnard, Paul Stewart, Hannah Walker, Doreen Rosenthal

**Affiliations:** Centre for Health Equity, Melbourne School of Population and Global Health, The University of Melbourne, Level 4, 207 Bouverie Street, VIC 3010 Melbourne, Australia

**Keywords:** Research practice, Indigenous research, Participants’ motivations and decision making, Research ethics

## Abstract

**Background:**

It is important for researchers to understand the motivations and decision-making processes of participants who take part in their research. This enables robust informed consent and promotes research that meets the needs and expectations of the community. It is particularly vital when working with Indigenous communities, where there is a history of exploitative research practices. In this paper, we examine the accounts of Australian Indigenous and non-Indigenous research participants in terms of how and why they agree to take part in research.

**Methods:**

A qualitative research approach was employed to undertake individual interviews with 36 research participants in Victoria, Australia. Eight participants identified as Indigenous and 28 were non-Indigenous. Thematic analysis was used to interpret the data.

**Results:**

There were stark differences between Indigenous and non-Indigenous research participants in terms of why and how they decided to participate in research. For Indigenous participants, taking part in research was primarily to benefit their communities rather than for personal interests. Indigenous participants often started from a position of caution, and showed a considered and deliberate process of decision making. In weighing up their decision to participate, some Indigenous participants clearly articulated what was valued in conducting research with Indigenous communities, for example, honesty, reciprocity, and respect; these values were explicitly used to assist their decision whether or not to participate. This was in contrast to non-Indigenous participants who took researchers’ claims on face value, and for whom deciding to participate in research was relatively straightforward. The motivations to participate of non-Indigenous participants were due to personal interests, a desire to help others, or trust in the medical practitioner who recruited them for the research project.

**Conclusion:**

Understanding research participants’ motivations about taking part in research is important. This is particularly relevant for Indigenous communities where there is a reported history of research abuse leading to mistrust. This understanding can lead to research practice that is more respectful and responsive to the needs of Indigenous communities and abides by the values of Indigenous communities. Moreover it can lead to more ethical and respectful research practice for all.

## Background

Understanding how and why research participants decide to take part in research is important for several reasons. Firstly, the capacity to recruit participants is obviously crucial to the conduct of research, and understanding participants’ motivations to participate can lead to more successful recruitment strategies. Secondly, understanding the processes of decision-making used by research participants, including the factors which they take into account and the way they approach the decision, is important in giving participants every opportunity to make an autonomous, informed choice about participation. This ensures a solid ethical foundation for research. Thirdly, understanding what is important to potential participants in research can assist in doing research that is relevant to, and addresses, the needs of the communities we engage with. This is particularly the case with communities that are traditionally under-represented and cautious about engaging in research.

Why and how participants decide to take part in research is especially vital when working with Indigenous peoples and communities where there is a history of exploitative and harmful research practices. This history has two legacies. Firstly, it has led to widespread mistrust of research among Indigenous communities world-wide [1–6] and an understandable caution on the part of Indigenous people to take part in research. This matters because good research is needed for improvements in health and service delivery in Indigenous communities. The second legacy is the obligation that this history imposes on current non-Indigenous researchers to do better. We propose that a better understanding of the reasons for participation and the process of decision making can lead to research practice that is more respectful of Indigenous communities and more responsive to their needs.

In this paper we discuss the motivations and reasons for participation given by people who have participated in sensitive social health research, focusing on the similarities and differences between Australian Indigenous^1^ and non-Indigenous research participants. Although there is considerable literature on why non-Indigenous participants agree to take part in research, there is much less empirical material reporting how Indigenous participants decide to participate in research and the reasons for their participation. This paper seeks to address that gap.

### Why non-indigenous participants agree to take part in research

There have been a number of studies that have examined the motivations of participants for taking part in research, particularly in health research [7–9]. These studies have primarily focused on non-Indigenous participants. A key reason why participants say they agree to take part in research is to help others and for broader societal benefit [10–13]. In addition to helping others, participants reported that they participated in research for personal benefits. These benefits can take many forms and includes gaining knowledge about a personal condition [13]. Studies investigating motivations of participants taking part in clinical trials report the potential to improve their own health [14], or to gain additional treatment or specialist attention [15, 16]. In the case of genetics research, another motivating factor for people participating in research is familial benefit, where future generations of the participants’ family could benefit from the potential genetics knowledge gained from the research [17].

Other forms of personal benefit reported include the opportunity to vent [11] and to be heard. The role of financial incentives has also been explored, with a number of scholars exploring practices of using reimbursement for participation in research [12, 18, 19]. From a review of relevant studies, Tishler et al. [20] found that financial payment was an important incentive for normal healthy volunteers in their decision to participate in clinical trials. However, in examining reasons for research participation, Hallowell et al. [17] caution us not to rely on simple, individualised ideas of why people decide to take part in research. In their examination of participation in cancer genetics research, Hallowell et al. [17] argued that participants gave personal, social and familiar reasons for participating. However, these reasons were interdependent and needed to be understood in relation to one another.

### Indigenous people’s participation in research

The long history of Indigenous communities being subject to unethical behaviour and approaches by researchers is well documented [5]. In Australia, health and medical research, as well as anthropological research, has often been conducted on Indigenous people primarily for the benefit of the non-Indigenous researchers, with little or no consultation or benefits for the communities involved. The research often adopted a ‘helicopter’ approach, with researchers entering the community for short periods of time, collecting and taking data away, with no further contact with the community. This has cemented a mistrust of researchers by Indigenous people [21]. There are similar patterns in other countries where research conducted in Indigenous communities has not been shared with, or returned to, those communities [22]. In some instances, research data has been taken, then subsequently used without permission, resulting in extreme breaches of trust [23]. These experiences have left Indigenous people suspicious and mistrustful of research and researchers [24, 25].

Processes of colonisation have resulted in vast health inequities for Indigenous people and communities throughout the world [26]. It has been recognised that in order to improve the health and wellbeing of Indigenous people and communities, there is an imperative to increase Indigenous participation in health and medical research [27–29]. An increasing amount of research now seeks to actively involve Indigenous people [30–33]; guidelines have been developed in order to facilitate respectful and productive relationships between researchers and Indigenous communities [34–36].

This paper builds on existing knowledge about research participants’ motivations and process of decision making with regard to research participation. We conducted individual interviews with Indigenous and non-Indigenous research participants and examined differences between these two groups in terms of why and how they decided to participate in research. Following a discussion of the methods employed, we present findings from both Indigenous and non-Indigenous research participants.

## Methods

This report is based on a larger qualitative research study investigating both participants’ and researchers’ understandings of trust in the research process. We gained ethical approval from the University’s Human Research Ethics Committee (HREC: 1034459) and all participants provided written informed consent. For this research we purposely recruited participants involved in ‘sensitive’ research. By focusing on sensitive research, the aim was to have a sample of people who had participated in research where, to them, the stakes regarding deciding to participate were at least moderately high. We defined ‘sensitive’ research in two ways. Firstly, we used the categories of participants identified in Australia’s *National Statement on Ethical Conduct in Human Research* [37] that were perceived to be vulnerable or required specific ethical attention. This included research with young people, and people in dependent or unequal relationships. Secondly, we defined ‘sensitive’ research to include research topics that touched on matters likely to be private or sensitive for the particular participants involved. To protect the privacy of our researcher-participants, whose names are publicly associated with their research projects, we do not identify particular research projects or topics in this paper. However, the research projects (that is, the ‘primary research projects’ from which we recruited both researchers and participants) broadly encompassed women’s and men’s social and health studies, and research with young people. We purposely included projects that used different methodological approaches to capture a broad range of participation experiences. Methodologies included surveys (paper and computer-assisted telephone interview [CATI]) and in-depth interviews, in both once-off and longitudinal studies. None of the primary research projects involved drugs, invasive procedures or biological specimens.

The study was specifically designed to include Australian Indigenous research participants and Indigenous researchers. This was based on the view that understanding of Indigenous perspectives is particularly important, partly because of the history of Indigenous research described previously, and also because unique perspectives from Indigenous people can shed light on the practices and attitudes of the broader community. This approach is consistent with the Australian guidelines for Aboriginal and Torres Strait Islander research, which state that: “Researchers should consider the application of their general research for the benefit of Aboriginal and Torres Strait Islander Peoples and the implications of cultural difference for its conduct” [34].

Recruitment of research participants began by first recruiting researchers who had conducted ‘sensitive’ research. When researchers were recruited to participate in this project, they were asked if we could also access and recruit their research participants at the completion of the data collection of the primary research project (we refer to the research study from which we recruited participants as the primary research project). Only researchers who granted us permission to access their participants were included in the sample. Researchers were not told which of their participants agreed to take part in our research. We sent letters of invitation for our project to potential research participants, together with information about the project and relevant contact details. Participants who accepted the invitation were then invited to take part in an individual, in-depth interview with a member of our research team. For Indigenous research participants, we used additional Indigenous-specific recruitment strategies. Recruitment was carried out in person or under the leadership and supervision of P, an Indigenous researcher in our research team. P is well known and respected in the local Indigenous community, with long experience in community-based research. Recruitment for Indigenous participants included working through Indigenous community organisations with which P has established collaborative relationships, and personal networks, as well as snowballing from those Indigenous people who initially agreed to participate. Despite these efforts, only a small number of Indigenous participants were able to be recruited.

In this paper, we focus on research participants, and specifically examine their motivations and decisions to participate in research. Our sample comprised 36 research participants (24 women and 11 men), aged between 18 and 70 years. Of these, 28 were non-Indigenous and 8 identified as Indigenous (Table 1).

**Table 1:** Sample of research participants

Project	Number of participants interviewed
Total non-Indigenous participants	28 (19 F, 9 M)
Total Indigenous research participants	8 (6 F, 2 M)
TOTAL research participants	36 (24 F, 11 M)

The majority of the interviews were conducted face-to-face, with one telephone interview for purposes of convenience for the participant. All interviews with Indigenous participants were face-to-face; most (approximately two-thirds) were conducted by P himself, with the remainder conducted by another member of the research team, as negotiated with the participant. The interviews for Indigenous and non-Indigenous participants were 30–60 mins duration and were audio-recorded for transcription, with participants’ permission. All interviews were conducted in English, the first or preferred language of all participants. Participants were asked about their previous involvement in research; their motivation for taking part in the original research project; their understanding of trust in research; their experiences of research participation and their overall perception of the research process.

The data collected from interviews were analysed using thematic analysis [38]. This method of analysis results in the generation of common themes and provisional hypotheses from the data. The data from the interview transcripts were organised into a system of coded patterns and themes. As well as analysing individual interviews, we conducted a comparative analysis across the interviews to look for any similarities and differences, particularly between the Indigenous and non-Indigenous research participants. The themes were systematically checked across each of the transcripts by different members of the research team to ensure their validity.

## Results and discussion

In the following sections we first discuss participants’ reasons for taking part in the primary research study, followed by their process of decision making. Within these sections, findings from the perspective on non-Indigenous research participants are contrasted with those of Indigenous participants.

### Participants’ reasons for taking part in the primary research study

#### Reasons for participation of non-Indigenous research participants

Turning first to non-Indigenous participants’ reasons for taking part in the primary research project, a number of reasons were offered. The first reason was a personal interest in the topic.

It’s because I’ve got the syndrome, I want more information about it. So when people come and ask me about it, I can give them the right information. And also, it also gives me… I learn things about it all the time. (Non-Indigenous Participant #25)

It is important to note that this was an individual interest, where participants either had the particular condition under investigation, or had some form of personal connection with it.

Second, participants had a desire to contribute to knowledge. This sense of altruism from participants is illustrated by the following:

The research seemed worthwhile and (was) contributing to knowledge. I’m hoping that my little piece somewhere along the line would help somebody else. (Non-Indigenous Participant #16)

So I thought if I participate in [primary project] the information will be collected and there will be an outcome. And people will be educated, and with education comes freedom and knowledge and you know, so that’s why I participate in these sort of things. (Non-Indigenous Participant #12)

This was a form of altruism, or desire to help others. The participants viewed the knowledge that would be generated as helpful to others in the future. As noted earlier, altruistic motivations for agreeing to participate in research are commonly reported in the literature.

For the following participant, a desire to help others was linked to having “no reason to say no”. The participant went on to say:

I guess I don’t mind participating in research and sometimes, when you’re on the spot like that, it’s just easier to agree sometimes to be honest, than to say – like I had no good reason to say no is the real reason and I don’t mind helping out people with their studies. (Non-Indigenous Participant #23)

Rather than altruism, this can be understood as a default disposition in favour of saying yes when asked to help others, when there is no great cost to self in terms of time, effort or risk. It is similar to the “why not” response that other researchers have previously reported [39, 40]

The third reason participants gave for agreeing to take part in the primary study was trust in the General Practitioner (GP) who recruited them to the primary study on behalf of the study investigators^2^:

I never felt pressured or anything like that, never felt pressured and because I’ve already got a very good relationship with that GP based on trust. As it is I didn’t ever feel that he would suggest something or send me info on something he didn’t think was trustworthy. (Non-Indigenous Participant #13)

Trust relationship was probably - initially the GP … [I]f it was just anybody I wouldn’t have done it because I wouldn’t know where the information was going. Because I knew who he [GP] was, he told me what it was about and what the purpose of what it was for, that’s why I, you know, was going to do it. (Non-Indigenous Participant #18)

It is interesting that these participants trusted that if the request was from their GP, then it was worthwhile for them as individuals to participate. For these participants, it did not appear that their motivation to participate was a particular interest in the research project. Rather it appeared to be triggered by a request from a trusted GP.

#### Reasons for participation of Indigenous research participants

All eight Indigenous research participants stated that their main reason for taking part in the primary (Indigenous) research was that it would benefit their communities.

That’s my community, I want to give something to my community. Therefore I will participate in this because I’m supporting my community. I’m part of the collective, it makes me feel good about my identity. (Indigenous Participant #30)

I guess I was interested in how we do research, and I’m much more interested in the translation of research and how that works for the community so they not only see what the research question is but how that improves things for the Aboriginal community. (Indigenous Participant #33)

I’m more happy to engage with [research] if it’s done locally for us. (Indigenous participant #29)

In a similar vein, one Indigenous participant reported taking part in research to help and support the Indigenous researcher.

Sometimes I’ve participated in research just because I wanted to support the person to get ahead with their life so you know like I was interviewed once by, you know someone who was doing their Masters, an Aboriginal person. I wasn’t really all that interested in being interviewed but I thought it’s good for their, you know I’m supporting them to get somewhere. (Indigenous Participant #30)

This quote reinforces the point that participation was not due to personal interest in the topic, but rather for the benefit of a fellow community member.

Although these participants cited the importance of community benefits in their motivation to participate in the primary research project, this was not a naïve exclusion of other considerations. Additional factors such as time required, personal risk and convenience were considerations to their participation. For example, Indigenous participants in a demographic study were comfortable contributing certain personal information, such as their weight and height but were reticent about the blood tests required as part of the study and declined to participate in that part of the study. As the following participant stated:

If I feel like I’ve got something to offer a project and they need an Aboriginal voice then I’ll agree to be in it. But … I don’t just willy nilly go in every research project. (Indigenous Participant #33)

A second reason given by some Indigenous participants was that they believed that they had something to contribute. Indigenous participants believed that their participation would contribute to an evidence base for the community, or would document an aspect of Indigenous community life that would otherwise be lost. This is illustrated by this participant’s response:

One, I think it’s important to get the facts. I get a sense that sometimes people rewrite history and rewrite it, sometimes it’s not accurate you know. Sometimes it’s more interpretations rather than the facts. There is history and there are perceptions of history and I just think that particularly for [Indigenous organisation] that we get it right and we get it right once. So that’s, that was my motivation. You know like the other thing is I don’t want anyone to rewrite my history. (Indigenous Participant #26)

In summary, the motivations of Indigenous research participants taking part in the primary research project were primarily to benefit their community, either directly or indirectly by contributing to knowledge for, or about, the community; however, this was not a result of a naïve exclusion of personal considerations.

### Participants’ process of deciding whether or not to take part in research

We were interested in participants’ process of decision making in relation to their participation in the primary research. We asked what they considered in deciding whether or not to participate. As with the previous section, we first examine the responses of non-Indigenous participants before contrasting this to the Indigenous participants.

#### Process of decision making for non-Indigenous participants

For non-Indigenous participants, there were two main themes identified: there were those who showed very little deliberation before deciding to take part, and those who did weigh up their decision, but their process of decision making did not appear very deliberative.

For most non-Indigenous participants, there was little thought given to deciding whether or not to participate:

At the time I didn’t really think much of it, I just thought I would try out – I sort of just thought I would help. (Non-Indigenous Participant #10)

For these participants, the decision-making process did not appear to involve an in-depth level of weighing up of risks and benefits, or serious considerations of researcher aims and motivations.

I was just signing up and just sort of did it; there was no real thought process as to maybe I should– just did it, that was about it. (non-Indigenous Participant #19)

All I thought is they want to know some answers to some questions and I’ll help, that’s it. That’s, that’s all that really – they asked me to help, so I helped, that’s it (Non-Indigenous Participant #24)

For other non-Indigenous participants, there was some thought given to whether or not they should participate, but they felt that they could trust the researcher and what he or she said.

I take it on face value that what they’re saying to me is the truth, so I guess if they have told me certain things about the project then I would take that at face value; and if something happened later and I found that sort of my name may have been attached to the results or the results were used in a way that hadn’t originally been sort of outlined to me then it would have an impact on trust. But I don’t know if anything could happen, sort of during the interview; it’s a bit hard to, to be worried about what people say when – you do have to trust that they are being truthful to you. (Non-Indigenous Participant #20)

Although this participant was prepared to accept the researcher’s claims on face value, the participant did consider the impact of a loss of trust if things went wrong. Nonetheless, their process for deciding whether or not to take part was relatively straightforward and did not seem to involve much reflection, deliberation, or weighing up of pros and cons.

There were participants who did weigh up their reasons for participating, as illustrated by the following comments:

I’m not a very trusting person naturally, but I think … it was more that I wanted to be involved in the study because I thought the topic was important enough to sort of outweigh any hesitation I might have had. (Non-Indigenous Participant #16)

This participant considered their natural lack of trust against the perceived importance of the research topic to them; although a process of weighing up occurred, this did not appear to be very deliberative or considered.

#### Process of decision making for Indigenous participants

In contrast to the non-Indigenous participants, the Indigenous participants were notable for their thoughtful and reflective discussion of their process of decision making before agreeing to participate in research. The following lengthy quote typified the responses of Indigenous participants with regard to the process of decision making.

We’re checking them [researchers] out as you would expect, we’re checking out how are they, how do they, what are they looking at when they look at us, you know. First thing we want to hear, clear in our head, is what’s their motivation, you know, what’s their agenda. You know we tend to be a little bit suspicious at times and sometimes for good reason so you know they’re the first things; and if somebody’s arrogant or rude or disrespectful or we think that there’s not … if we think there’s something going on, then it’s over… But to [primary researcher’s] credit, he – maybe it’s a little bit of personality based - but he came across as pretty honest. He was prepared, he was transparent, and he didn’t say this is what I want, you know. He said this is what we can do, you know - so it was all, it was good conversations and he promised things that he did, and when he promised things he did them.

…We’ve had researchers come, come across with their ideas and their perceptions and I had one the other day which was a really interesting project which you know I’m just turning it over in my head whether the outcomes could be really good for us, but the approach so far hasn’t been good. (Indigenous Participant #26)

Here this participant clearly spelled out their expectations and what factors they weighed up when approached to take part in research; these included the researcher’s motivations (their agenda), as well as their level of honesty and respect. During the process of research, the scrutiny continued, with the participant assessing whether or not the researcher fulfilled their promises. Clearly for this participant, just being interested in the research project was not enough justification for agreeing to participate. Of note in this quote is the participant’s consistent use of ‘we’ and ‘us’; this strongly reflects the reason cited by Indigenous participants of the requirement for the Indigenous community to benefit as the basis for research participation.

In the quote above, the participant is referring to a non-Indigenous primary researcher. For most Indigenous participants, when the researcher was also Indigenous, there was a base of shared understanding and expectations. The following quotes point to the shared understanding and trust, not just with other Indigenous researchers but also with Indigenous organisations involved in research:

I think with the (primary) project, you knew that it was an Aboriginal organisation that was doing the project so you sort of had that trust, because you knew who was doing it. Whereas some research projects, you’re really not sure who is doing it, what their motives are and whether that’s either the individual researcher, if it’s to sort of, to further their career or if it’s just research for research sake or I don’t know… I think it’s something you have to consider more. (Indigenous Participant #34)

This shared understanding and expectations of what was expected when conducting research with Indigenous communities was prevalent in all interviews with Indigenous participants.

Well there are protocols, we’ve got protocols. I mean you can go to uni all you like, but when you come into our community there are protocols and you’ve gotta tread slowly and it’s almost like a list of these [protocols]: this is how you engage with us. If you can’t embrace that, if you’ve got something in your head that ‘no, I don’t wanna follow them rules’, the relationship’s over or it’ll never be as good as what it could be. And there are non-Aboriginal people who embrace them protocols about respect, being good listeners, don’t promise what you can’t deliver, don’t build up expectations, don’t try to get info cheap, don’t think we’re gonna do it for nothing. You know they just, there’s no problem about that type of thing. Give us feedback, be honest. (Indigenous Participant #26)

In this quote, this participant detailed the protocols to be followed when undertaking research with Indigenous communities: listening, not promising unrealistic outcomes, acknowledging participation, honesty, reciprocity through providing feedback, and respect for communities. What is noteworthy here is the sophisticated understanding and clarity in describing what is valued in conducting research with Indigenous communities, and what factors community members will take into account when deciding whether or not to agree to participate in a particular research study. This was in contrast to the many non-Indigenous participants whose decision to participate in research was made readily without considering a range of possible concerns.

Unlike the non-Indigenous participants who took being asked to participate in research at face value and started from a position of assumed trust, Indigenous participants appeared to begin from a position of caution and distrust, and researchers had to earn their trust:

I’m more suspicious of researchers I don’t know. …I’ve been a member of [name of professional association]; there’s been a lot of stuff around researchers and trust, and I guess from my point of view …you know, they have to earn their trust. (Indigenous Participant #33)

This starting point of suspicion is understandable in context of the history of unethical research practices in Indigenous communities, as noted earlier. The following quote from an Indigenous participant highlights this abuse.

One of my bug bears is data mining…Researchers collect all this health information… and they keep re-using that data and not informing the participants and I just think that’s quite abusive, that the participants aren’t fully informed that their data’s going to be looked at and analysed like ten or twenty times and produce new things. Where the researcher benefits from it but the community doesn’t … To me, that’s sort of abusive, if we just keep defining the problem and not doing anything about it. (Indigenous Participant #33)

Other considerations for Indigenous participants taking part in research were privacy and confidentiality. Not only were these issues part of the weighing up process in deciding whether or not to actually participate, they continued to be deliberated upon during the process of data collection:

And I just thought no, I think this is not what I want to share and I have my own reasons; and it’s part of, you know, making sure my community’s alright, myself and that they go and talk to whoever it is they need to talk to about stuff. (Indigenous Participant #32)

It is because you’re trying to work out how much do they [researchers] need to know, and how much is appropriate to give them, and what impact it has in that space. (Indigenous Participant #35)

When confidentiality was properly observed during the conduct of the research, this was duly noted.

He [Researcher] said I will send you your information only, I can’t send you anyone else’s, that’s all confidential and we said, that’s good cos we understand that confidentiality stuff, so that was good that he reinforced that. So my story hasn’t gone to anyone else but I’ve got the document, he mailed it to me. (Indigenous Participant #26)

In summary, Indigenous research participants’ motivations to participate were governed by community rather than personal interests, and their process of decision-making was far more considered and deliberative than the non-Indigenous participants. Non-indigenous participants either had personal motivations for agreeing to participate in research, or had very broad, generalised altruistic motivations. These motivations were against a background of perceived unreflective trust in researchers, and a pre-disposition to say yes, or ‘why not’ to research that was not obviously too costly in time, effort or risk for them.

## Conclusion

Overall, we found considerable differences between Indigenous and non-Indigenous participants about why and how they decided to participate in research. For Indigenous participants, the motivation to participate was primarily due to benefits to their community, rather than personal interest or benefit, a key motivator for the non-Indigenous participants. Non-Indigenous participants certainly did cite helping others as an important reason for agreeing to participate in research. However, this sense of altruism was quite vague and general, whereas Indigenous participants clearly had their own community in mind. Indigenous participants demonstrated considered and deliberative processes, assessing the research and researcher against well-articulated ‘protocols’. This was in contrast to non-Indigenous participants who were more likely to take researchers at face value, or did not appear to engage in-depth assessments.

It is interesting that the process of decision-making described by Indigenous participants very closely matches the research ethics ideal of informed consent, which envisages a rational deliberative process in which people gather information, and use that to carefully weigh up the pros and cons before agreeing to participate [41]. In contrast, non-Indigenous participants appeared to do very little weighing up and it is less clear that they actually gave truly informed consent in the standard ethical sense. As a concept and an ethical value, informed consent is often criticised as an individualistic notion arising from western culture [42, 43]. So it is noteworthy that here, our Indigenous participants described undertaking a process very like informed consent to manage their interactions with non-Indigenous researchers. One difference from the standard model of informed consent is that the information that Indigenous participants are using is not the information that is typically given in a written Participant Information Statement. Rather, it is information which they have gathered themselves by talking with and observing the researcher, sometimes before any research has formally commenced. Thinking about this kind of decision-making process as informed consent opens up the possibility of a more active, information gathering model of informed consent as a possibility for all research participants.

We suggest that these findings are important for researchers working with Indigenous communities, especially non-Indigenous researchers. Understanding what motivates Indigenous people to participate in research and their process of decision making can assist researchers to plan and conduct their research in a way that will be ethically appropriate and practically feasible. The key concerns of Indigenous people are that their communities will likely benefit from the research, and that researchers are respectful, honest and committed. These factors should be taken into account by researchers from the outset. Researchers can expect a process of being ‘checked out’ by prospective Indigenous participants; understanding the reasons behind this means that researchers can prepare and engage respectfully in the process.

It is important to note that the Indigenous participants in this study may have particular characteristics, views and experiences not shared by other Indigenous people who have participated in research. Despite considerable effort put into recruitment, we were only able to interview eight Indigenous research participants. We can only speculate on the reasons for this which may include a perception that the research would not directly benefit their community, lack of trust in the institution (a large research university) even if there was a sense of trust in individual researchers, and a sense of participation burnout, in addition to other personal and time commitments. Although we acknowledge that the small sample size is a limitation of the study, we formed the view that it was nonetheless important to report on these participants. There was a high level of consistency in their responses, and they were particularly articulate and thoughtful in what they said. They may well have agreed to be interviewed for our study because they had a view which they wanted heard, especially given their account that the consideration of benefit to their community is one of the main drivers of their participation in any research. However, this in no way invalidates the findings. Their responses are indicative of a clearly-formed and strongly held position on research participation in the Indigenous community in our region. We argue that there is much to learn from this, even if we cannot say definitively that this is the only or the predominant position held by all Indigenous Australians.

The legacy of the history of research abuse of Indigenous peoples is clearly still pervasive in Indigenous communities; historically, non-Indigenous researchers did not consult with Indigenous communities, were disrespectful and were primarily concerned with their own interests rather than a sense of reciprocity or benefit to the community. To enable research that strives towards health equity in Indigenous communities it is important to understand, from the perspectives of Indigenous people themselves, why they take part in research and how they decide to do so. We need to be aware that the motivations and decision-making processes of non-Indigenous research participants are not necessarily applicable in Indigenous settings. This, combined with recent documented studies on how to conduct meaningful Indigenous research participation [33], provides a sound base for thinking about ways to conduct research with Indigenous communities that is respectful and meaningful for them. Resources are available to guide the ethical conduct of research with Indigenous communities. Notably in Australia there are the *Values and ethics: Guidelines for ethical conduct in Aboriginal and Torres Strait Islander health* research [34]. There are six values that form the foundation of these guidelines: Spirit and Integrity; Reciprocity; Respect; Equality; Survival and Protection; and Responsibility. Of interest is the close alignment of one Indigenous participant’s discussion of the ‘protocols’ with these guidelines. The practices advocated by this participant of listening, not promising unrealistic outcomes, acknowledging participation, being honest, providing feedback, and showing respect for communities and land, have these six values as their base. We have both the guidelines and the perspectives of Indigenous people to inform our research practice; we suggest that it is now a matter of putting these into action.

## Footnotes

1 The term ‘Indigenous’ is used within this paper to refer to the Aboriginal and Torres Strait Islander people of Australia. We acknowledge that Aboriginal and Torres Strait Islander people may identify with their clan, skin, tribe and/or geographical name and we mean no disrespect in using this term.

2 For these projects, the GP was only responsible for recruiting participants, and was not actually part of the primary research team or our research team.
